# Regulators of *Slc4* bicarbonate transporter activity

**DOI:** 10.3389/fphys.2015.00166

**Published:** 2015-06-12

**Authors:** Ian M. Thornell, Mark O. Bevensee

**Affiliations:** ^1^Department of Cell, Developmental and Integrative Biology, University of Alabama at BirminghamBirmingham, AL, USA; ^2^Nephrology Research and Training Center, University of Alabama at BirminghamBirmingham, AL, USA; ^3^Center of Glial Biology in Medicine, University of Alabama at BirminghamBirmingham, AL, USA; ^4^Civitan International Research Center, University of Alabama at BirminghamBirmingham, AL, USA

**Keywords:** acid-base, anion exchanger, cotransporter, pH, signaling

## Abstract

The *Slc4* family of transporters is comprised of anion exchangers (AE1-4), Na^+^-coupled bicarbonate transporters (NCBTs) including electrogenic Na/bicarbonate cotransporters (NBCe1 and NBCe2), electroneutral Na/bicarbonate cotransporters (NBCn1 and NBCn2), and the electroneutral Na-driven Cl-bicarbonate exchanger (NDCBE), as well as a borate transporter (BTR1). These transporters regulate intracellular pH (pH_i_) and contribute to steady-state pH_i_, but are also involved in other physiological processes including CO_2_ carriage by red blood cells and solute secretion/reabsorption across epithelia. Acid-base transporters function as either acid extruders or acid loaders, with the Slc4 proteins moving HCO^−^_3_ either into or out of cells. According to results from both molecular and functional studies, multiple Slc4 proteins and/or associated splice variants with similar expected effects on pH_i_ are often found in the same tissue or cell. Such apparent redundancy is likely to be physiologically important. In addition to regulating pH_i_, a HCO^−^_3_ transporter contributes to a cell's ability to fine tune the intracellular regulation of the cotransported/exchanged ion(s) (e.g., Na^+^ or Cl^−^). In addition, functionally similar transporters or splice variants with different regulatory profiles will optimize pH physiology and solute transport under various conditions or within subcellular domains. Such optimization will depend on activated signaling pathways and transporter expression profiles. In this review, we will summarize and discuss both well-known and more recently identified regulators of the Slc4 proteins. Some of these regulators include traditional second messengers, lipids, binding proteins, autoregulatory domains, and less conventional regulators. The material presented will provide insight into the diversity and physiological significance of multiple members within the *Slc4* gene family.

## Introduction

The *Solute carrier 4* (*Slc4*) gene products are part of a family of bicarbonate transporters (BTs) that include Na^+^-independent anion exchangers (AEs), as well as Na^+^-coupled bicarbonate transporters (NCBTs). NCBTs are further categorized into (*i*) Na/bicarbonate cotransporters (NBCs) that are either electrogenic (NBCe1 and NBCe2) or electroneutral (NBCn1 and NBCn2), and (*ii*) the electroneutral Na-driven Cl-bicarbonate exchanger (NDCBE). BTs regulate intracellular pH (pH_i_) and can alter extracellular pH (pH_o_) by functioning as either acid loaders that transport HCO^−^_3_ (or CO^2−^_3_) out of cells, or acid extruders that transport these ions into cells.

BTs also contribute to the cellular homeostasis of other ions and associated properties in addition to H^+^ and HCO^−^_3_. For example, the Cl-HCO_3_ exchanger (Cl^−^ into cells, HCO^−^_3_ out) can act in concert with the Na-H exchanger (Na^+^ in, H^+^ out) to move net NaCl into cells, thereby increasing intracellular Na^+^ (Na^+^_i_) and Cl^−^ (Cl^−^_i_), but with no net movement of acid-base equivalents. Activating this pair of transporters in shrunken cells promotes cellular water uptake in a process known as volume regulatory increase (Hallows and Knauf, 1994). As another example, NCBTs contribute to the regulation of Na^+^_i_ and intracellular Ca^2+^ (Ca^2+^_i_), and can directly influence membrane excitability. Such regulation is particularly evident in the heart where there is both electroneutral NBC activity (Dart and Vaughan-Jones, 1992; Lagadic-Gossmann et al., 1992; Leem et al., 1999) and electrogenic NBC activity (Camilión de Hurtado et al., 1995, 1996; Aiello et al., 1998). During ischemia and subsequent reperfusion of the heart, Na-coupled acid-base transporters in myocytes that are activated by ischemia-induced intracellular acidosis can elevate Na^+^_i_, which in turn can decrease and even reverse the Na-Ca exchanger, thereby elevating Ca^2+^_i_and promoting Ca^2+^-mediated injury. Indeed, NBC activity contributes to the pH_i_ recovery of heart cells with reperfusion following ischemia/hypoxia (Vandenberg et al., 1993; Schafer et al., 2000; van Borren et al., 2004; Ten Hove et al., 2005; De Giusti et al., 2011; Fantinelli et al., 2014), but can also promote damaging Na^+^_i_ and Ca^2+^_i_ overload (Schafer et al., 2000; Ten Hove et al., 2005). An antibody reported to inhibit human-heart NBCe1-B can protect the systolic and diastolic functions of rat heart during reperfusion (Khandoudi et al., 2001). However, cardioprotection from NBC blockade is not always evident depending on experimental conditions (Ten Hove et al., 2005). Consistent with the findings by Khandoudi et al. (2001), an antibody targeting the third extracellular loop of NBCe1 that inhibits NBCe1-B (De Giusti et al., 2011) limited the infarct size and protected the systolic and diastolic functions of reperfused isolated rat hearts (Fantinelli et al., 2014). Curiously, the compound S0859, which inhibits multiple NCBTs in the heart (Ch'en et al., 2008), limited the infarct size, but did not protect the systolic and diastolic functions (Fantinelli et al., 2014). In addition to contributing to pH_i_ and ion homeostasis, electrogenic NBC activity produces an outward current that contributes to the resting membrane potential of cardiac cells and can shorten the action potential duration (Aiello et al., 1998; Villa-Abrille et al., 2007; De Giusti et al., 2011).

BTs not only contribute to ion homeostasis and steady-state pH_i_ of cells, but also promote solute secretion and/or reabsorption by epithelia. For example, NBCe1-A in the basolateral membrane of cells in the renal proximal tubule (Boron and Boulpaep, 1983) is responsible for up to 90% HCO^−^_3_ reabsorption by the kidney. Basolateral NBCe1-B contributes to HCO^−^_3_ secretion by the pancreas (Muallem and Loessberg, 1990). Apical NDCBE works in conjunction with the apical anion exchanger pendrin to account for thiazide-sensitive NaCl reabsorption in the cortical collecting duct of kidney (Leviel et al., 2010). BTs also have roles ancillary to pH_i_ regulation and solute transport. For example, the erythrocyte AE1 (eAE1) is responsible for the chloride (Hamburger) shift in erythrocytes that facilitates CO_2_ carriage in the blood from systemic to lung capillaries. NCBTs such as NBCe1, NBCn1, and NDCBE elicit pH_o_ shifts in the nervous system that can modulate neuronal firing (Chesler, 2003).

Key advances in understanding the molecular nature of BTs came when Kopito and Lodish (1985) first cloned the cDNA encoding an anion exchanger (eAE1), and Romero et al. (1997) first cloned the cDNA of a NCBT (NBCe1-A). From additional cloning studies of other BTs, it became apparent that each family member has multiple variants that arise from alternative promoters or splicing. Nearly all of the variability occurs within the cytoplasmic amino (N)- and/or carboxy (C)- terminal domain(s) (Boron et al., 2009; Parker and Boron, 2013). These variable domains are potential targets for intracellular regulators that modulate specific transporter activity, thereby tightly controlling associated HCO^−^_3_ transport and pH_i_ changes. A regulator can alter a BT's transport activity by changing either transport biophysics (i.e., K_M_ and V_max_), transporter expression, or perhaps transport stoichiometry. Consequently, BT activity can be optimized for a given cell type depending on the BTs expressed and the signaling pathways and regulators either present or activated. There is considerable interest in understanding how *Slc4* family members and their variants are regulated, thereby providing insight into the role of these variants in normal and abnormal physiology.

In this review, we will present our current understanding of regulatory mechanisms of the cloned BTs. We will first briefly review the molecular physiology of BTs, with an emphasis on areas pertinent to cell signaling. We will then examine each family member with an emphasis on functional importance and specific modes of regulation.

### Molecular physiology

The BT family consists of 10 genes that encode Slc4 proteins, which can be divided into the following three groups: Na^+^-independent AEs, Na^+^-dependent anion exchangers (i.e., NCBTs), and a borate transporter. The AEs consist of AE1-4 (encoded by *Slc4a1-3,9*). AE1-3 are acid loaders that normally exchange 1 Cl^−^ into cells for 1 HCO^−^_3_ out of cells. At least AE1 can also mediate sulfate/H^+^ cotransport (Milanick and Gunn, 1984) and oxalate/H^+^ cotransport (Jennings and Adame, 1996) in exchange for 1 Cl^−^. The function of AE4 is not entirely clear, and there is evidence that the transporter may be Na^+^ dependent (Parker and Boron, 2013).

The electrogenic NCBTs include NBCe1 (*Slc4a4*) and NBCe2 (*Slc4a5*). Electrogenic NBCs are typically reported to cotransport 1 Na^+^ and either 2 or 3 HCO^−^_3_. Recently however, it has been discovered that CO^2−^_3_ instead of 2 HCO^−^_3_ appears to be the transported species for NBCe1 (Lee et al., 2011; Moss et al., 2014). CO^2−^_3_ transport into cells can have important consequences, and is predicted to lead to a rise in extracellular P_CO2_, as discussed for such transport in the brain (Grichtchenko and Chesler, 1994; Voipio, 1998; McAlear and Bevensee, 2004). This P_CO2_ increase occurs because extracellular HCO^−^_3_ is rapidly converted into more CO^2−^_3_ and H^+^ (pK ~ 10.4). However, because the H^+^ increase is disproportionately larger than the HCO^−^_3_ decrease, equilibration requires the two to react and form more CO_2_ and H_2_O. This production of extracellular CO_2_ and its subsequent rapid diffusion may be a valuable route for efficiently removing acid equivalents from metabolically active tissue (Voipio, 1998). For a 1:3 Na:HCO^−^_3_ stoichiometry, CO^2−^_3_ likely transports with a single HCO^−^_3_ (Zhu et al., 2013). For transport into cells, the energetically favorable inward electrochemical gradient for Na^+^ drives transport.

The remaining NCBT members that complete the family are electroneutral. *Slc4a7* encodes the electroneutral Na/HCO_3_ cotransporter (NBCn1), which normally extrudes acid by cotransporting 1 Na^+^ and 1 HCO^−^_3_ into cells. *Slc4a8* encodes the Na-driven Cl-HCO_3_ exchanger (NDCBE), which extrudes acid utilizing the electrochemical energy of 1 Na^+^ into cells to drive the exchange of HCO^−^_3_ into cells for 1 Cl^−^ out of cells. In the transport process, two intracellular acid equivalents are neutralized, and this is likely achieved by the transport of 1 CO^2−^_3_ (Grichtchenko and Boron, 2002). *Slc4a10* encodes the electroneutral Na/HCO_3_ cotransporter (NBCn2), which normally extrudes acid by cotransporting 1 Na^+^ and 1 HCO^−^_3_ into cells, but with an associated futile Cl^−^ self-exchange (Parker et al., 2008; Damkier et al., 2010). In early literature, this transporter was named a Na-driven Cl-HCO_3_ exchanger (NCBE). The transport mode of NBCn2 (NCBE) appears complex with a Cl^−^ dependence that may depend on transport direction, species of the transporter, and/or expression system (see Majumdar and Bevensee, 2010; Parker and Boron, 2013).

The remaining BT is the *Slc4a11*-encoded borate transporter, which is a Na^+^-coupled base transporter capable of Na-H exchange in mammalian cells (Jalimarada et al., 2013; Ogando et al., 2013; Kao et al., 2014). This borate transporter, along with AE4 described above, are the least functionally understood of all the BTs.

Structural information is available for BTs, particularly for AE1, but also for NCBTs that are 28-34% homologous to AE1 (Romero et al., 2004; Parker and Boron, 2013). The N-terminal 40% of AE1 is the cytosolic domain involved in binding other proteins and regulators, whereas the remaining 60% of the protein is the C-terminal membrane domain involved in ion transport (Reithmeier et al., 1996). Based on considerable data from proteolysis, labeling, and mutagenesis studies, AE1 is predicted to have 14 transmembrane domains with most spanning the membrane, and the N and C termini located in the cytosol (Cordat and Reithmeier, 2014). Using similar approaches and mapping onto the predicted topology of AE1, investigators have developed similar models for NCBTs such as NBCe1 (Zhu et al., 2010a,b; Kurtz and Zhu, 2013a,b; Parker and Boron, 2013), although there are likely structural differences among these transporters (Zhu et al., 2010a; Parker and Boron, 2013). A challenge in the field is to identify the structural similarities—or perhaps core structure—of BTs, and then the structural differences that define their functional uniqueness.

An early 3D crystal structure of the C-terminal membrane domain of AE1 resolved to 20 Å revealed a dimer that contains a cytoplasmic region that likely directs substrate to a membrane region for translocation across the membrane (Wang et al., 1994; Reithmeier et al., 1996). The structure of the N-terminal cytosolic domain of eAE1 resolved to 2.6 Å revealed an interlinking N-terminal dimerization site (Zhang et al., 2000), and a similar structure resolved to 2.1 Å of this domain missing its disordered regions was obtained at neutral pH (Shnitsar et al., 2013). The subsequent crystal structure of the dimeric C-terminal membrane domain at 7.5 Å supported these models, and also revealed a surprising similarity to the structure of the prokaryotic ClC chloride channel, which functions as a Cl-H exchanger (Yamaguchi et al., 2010; Hirai et al., 2011). These proteins have an inverted structural repeat of 5 transmembrane domains that is common among other transporters from different gene families including the Na/leucine cotransporter LeuT. More recently, Barneaud-Rocca et al. (2013) reported a similar homology model of the membrane domain of AE1 based on the crystal structure of the bacterial uracil/H symporter UraA, and provided evidence that specific transmembrane domains (e.g., 3, 5, and 8) form the ion-translocation pathway (Barneaud-Rocca et al., 2013). Cordat and Reithmeier (2014) provide a more detailed review of the aforementioned structures of AE1. Although a high-resolution crystal structure has not been solved for any NCBT, the N terminus of NBCe1-A resolved to 3 Å reveals an interlinking N-terminal dimerization site (Gill and Boron, 2006) similar to that for AE1. Because of the similarity between AE1 and NCBTs, the structures of ClC and UraA used to model AE1 will also be useful in developing structural models of NCBTs.

In this review, we focus on AE1, AE2, NBCe1, NBCn1, NDCBE, and NBCn2 because their modes of regulation are the best characterized to date. Alper et al. (2002), Romero et al. (2004), Parker and Boron (2013), and Cordat and Reithmeier (2014) provide a more in-depth discussion of BT family members beyond the scope of regulation.

## AE1 (*Slc4a1*) and AE2 (*Slc4a2*)

During the cloning era, one of the early cDNAs identified encoded the murine erythrocyte AE1 (eAE1) (Kopito and Lodish, 1985). eAE1 is commonly known as band 3 protein because it is the third band resolved when total erythrocyte protein is separated by gel electrophoresis. AE1 normally functions as an acid loader by exchanging 1 extracellular Cl^−^ for 1 intracellular HCO^−^_3_, but can transport in the opposite direction by changing the ion gradients (e.g., by removing extracellular Cl^−^) as described below for erythrocytes.

In erythrocytes, AE1 is an integral component of the Jacobs-Stewart cycle, which is responsible for increasing the total CO_2_-carrying capacity of blood from the systemic circulation to the lungs, and allowing large amounts of this CO_2_ to be carried in the form of HCO^−^_3_ (Boron, 2012). In brief, about two-thirds of CO_2_ that enters the erythrocytes is hydrolyzed to H^+^ and HCO^−^_3_— mainly through the action of carbonic anhydrase (CA). eAE1-mediated Cl-HCO_3_ exchange, which is also known as the Cl^−^ or Hamburger shift, prevents the rate-limiting accumulation of intracellular HCO^−^_3_ by transporting the HCO^−^_3_ out of the erythrocytes. AE1 also contributes to the CO_2_- and pH-dependent oxygen-carrying capacity of hemoglobin— also known as the Bohr effect. About 25% of total protein in erythrocytes is eAE1 (Fairbanks et al., 1971).

**Figure 1:**
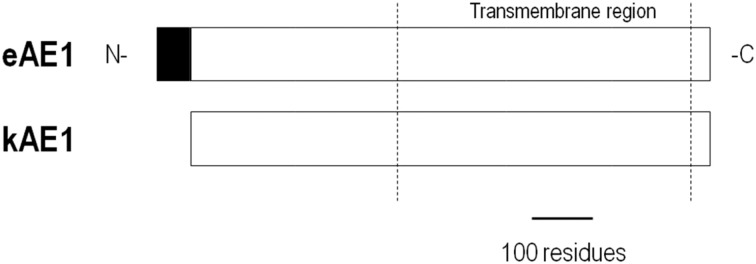
**Alignment of AE1 variants**. kAE1 is identical to eAE1 except for missing the N-terminal 65 residues.

AE1 is also densely expressed in the basolateral membrane of α-intercalated cells in distal nephron segments of the kidney (Alper et al., 1989). Kidney AE1 (kAE1) lacks the N-terminal 65-amino acid residues found in eAE1 (Figure 1). In the distal nephron, kAE1 promotes basolateral uptake of intracellular HCO^−^_3_ that is formed from apical H^+^ secretion for subsequent urinary excretion. AE1 mRNA has also been detected in the heart (Kudrycki et al., 1990; Richards et al., 1999) and colon (Kudrycki et al., 1990) where the protein's role is less defined.

**Figure 2:**
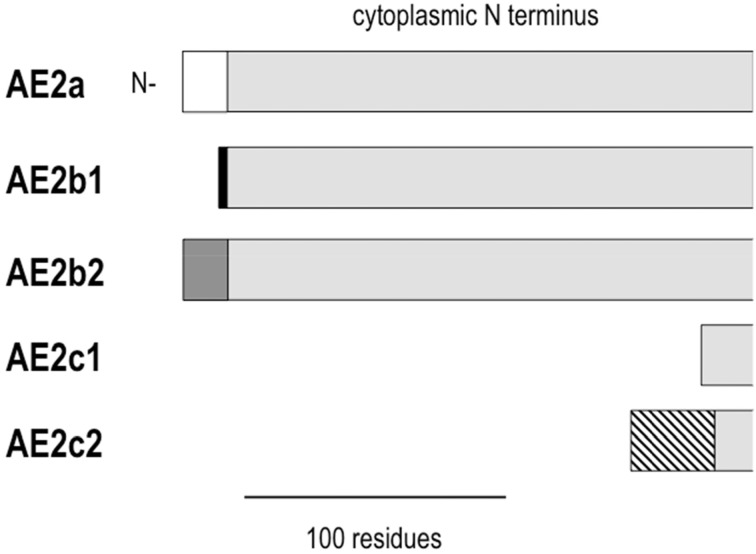
**Alignment of the cytoplasmic N termini of AE2 variants**. AE2 variants are identical except for differences at the N terminus. Common residues are shown in light gray. The different N termini are defined by 17 residues for AE2a (white), 3 residues for AE2b1 (black), 8 residues for AE2b2 (gray), a shortened N terminus for AE2c1, and 32 residues attached to a shortened N terminus for AE2c2 (hatched).

AE2 is found in the basolateral membrane of most epithelia. AE2 is functionally similar to AE1 in having a 1:1 Cl^−^:HCO^−^_3_ exchange stoichiometry. There are three AE2 variants (Figure 2) that derive from alternate promoters, and they are designated by the letters a, b, and c (e.g., AE2a). AE2b and AE2c each have two additional variants designated 1 (AE2b1 and AE2c1) and 2 (AE2b2 and AE2c2) that arise from either overlapping promoter sequences (for AE2b) or splicing (for AE2c). The N termini of the AE2 variants are much longer than the corresponding N termini of either AE1 variant. AE2a is ubiquitous among tissue types. AE2b is less ubiquitous and densely expressed in stomach tissue. AE2c is expressed exclusively in stomach tissue (Wang et al., 1996).

### Carbonic anhydrase

Carbonic anhydrase (CA) is an enzyme that catalyzes the net reversible reaction CO_2_ + H_2_O ↔ H_2_CO_3_, which is in rapid equilibrium with HCO^−^_3_ and H^+^. In the presence of CA, the rate of the hydration reaction is nearly limited by diffusion. It is clear that CA activity promotes the formation or removal of HCO^−^_3_ that can influence BT activity. Endogenous CA isozyme II (CAII) in HEK293 cells appears to keep AE1 maximally active based on the observations that AE1 is inhibited by either applying the CA inhibitor acetazolamide or co-expressing a dominant negative CAII, but unaffected by co-expressing wild-type CAII (Sterling et al., 2001). Sowah and Casey (2011) presented evidence that active CAII fused to the C terminus of AE1 stimulates AE1-mediated changes in intracellular pH and chloride. In this study, the dominant-negative effect of a catalytically inactive CAII implicates a physical interaction between CAII and AE1. In exploring the functional interplay between CA in AE activity, investigators have provided evidence that CAII directly interacts with AE1 to form a “metabolon” by binding to the short 33-residue C terminus of AE1. It has been proposed that the proximity of CAII to AE1 provides localized generation or removal of HCO^−^_3_ to adjust AE1 activity. However, binding of CAII to AE1, and its functional implications remain controversial.

There is experimental evidence that both supports and refutes a physical interaction between CA and AE. Results consistent with a direct interaction come from solid phase binding assays with purified CAII and a C-terminal 33-residue peptide of AE1 conjugated to GST (GST-AE1), which can be detected by ELISA using an antibody to GST (Vince and Reithmeier, 1998). GST-AE1 (10 nM) interacted with immobilized CAII under physiological conditions of pH and ionic strength, and the binding rate increased by either raising the GST-AE1 concentration or lowering the pH and ionic strength. Furthermore, an antibody to the C-terminal residues inhibited the CAII-AE1 interaction. The binding site within AE1 for immobilized CAII was identified as residues 887DADD in a mutagenesis study and based on the ability of mutant untagged AE1 constructs to compete with wild-type GST-AE1 for binding (Vince and Reithmeier, 2000). Results from additional early studies also provide support for a direct CA-AE interaction. For example, in the aforementioned CAII-AE1 study with HEK293 cells, mutating the putative CA binding domain of AE1 reduced the exchanger activity (Sterling et al., 2001).

However, results from more recent studies do not support CAII binding directly to the C terminus of AE1. In a binding assay similar to that described above, Piermarini et al. (2007) replicated the finding that the GST-tagged C terminus of AE1 bound to immobilized CAII to a greater extent than the GST alone, however untagged AE1 did not bind. When reciprocal experiments were performed with either immobilized AE1, GST-AE1, or GST, soluble CAII displayed undetectable binding to untagged AE1, and bound to the fusion protein with approximately half the apparent affinity as to GST alone. Using surface plasma resonance to investigate potential transient interactions, the authors found that the CAII inhibitor acetazolamide (positive control), but not the untagged AE1 C-terminal construct interacted with immobilized CAII (Piermarini et al., 2007). The group concluded that GST-tagged C-terminal AE1 constructs and GST itself in the mobile phase bind to immobile CAII, while non-tagged constructs do not. The authors did not rule out the existence of an AE1-CAII metabolon, but concluded that it would be independent of the putative C-terminal domain of AE1. Similar results were obtained with CAII and the putative CAII-binding domains at the C termini of NBCe1 and NDCBE (Piermarini et al., 2007). These results sound a word of caution when assessing results from binding assays using labeled protein.

More recent evidence against direct binding of CAII to AE1 was obtained by FRET analysis using tsA201 cells co-transfected with the optimized FRET pair CAII-CyPet and YPet-AE1 (Al-Samir et al., 2013). Using single-channel confocal microscopy to examine the cross-sectional distribution of each fluorophore-conjugated protein, the authors found that AE1 was predominantly expressed in the plasma membrane, while CAII was homogenously expressed throughout the cell. CAII-CyPet and YPet-AE1 did not interact based on FRET analysis. In positive-control experiments, FRET was observed in cells transfected with the following two constructs: AE1 conjugated to either YPet at the N terminus or CyPet at the C terminus.

### Glycophorin A

Glycophorin A (GPA) is a single membrane passing sialoglycoprotein of 131 residues that is enriched in erythrocytes (for a review, see Chasis and Mohandas, 1992). GPA contains antigenic determinants of the MNS blood group. Co-expressing AE1 with GPA —but not glycophorin B or C— enhanced both AE1 expression and AE1-mediated ^36^Cl^−^ uptake in *Xenopus laevis* oocytes (Groves and Tanner, 1992, 1994). Different regions of GPA are responsible for increasing AE1 expression and function. For example, mutating extracellular residues 61–70 of GPA decreased the activity of AE1, while mutating the cytoplasmic domain of GPA inhibited trafficking of the protein to the cell surface (Young and Tanner, 2003). Erythrocytes that lack eAE1 also lack GPA. These data are consistent with AE1 functioning as a chaperone-like protein and recruiting GPA to the plasma membrane, where it then stimulates the transporter (Hassoun et al., 1998). There appears to be a mutual dependency between AE1 and GPA for proper expression. As suggested by Groves and Tanner (1992), GPA influences AE1 biosynthesis, and may synchronize increased plasma membrane expression of active AE1 with maturation of the erythrocyte.

GPA stimulation of AE1 may account for the observation that some AE1 mutations cause recessive distal renal tubular acidosis and hemolytic anemia, but retain normal anion transport in erythrocytes. One such AE1 mutant, G701D, displays reduced expression and function (^36^Cl^−^ uptake) compared to wild-type AE1 when expressed in oocytes (Tanphaichitr et al., 1998; Young and Tanner, 2003). However, co-expressing GPA increased both expression and function of the G701D mutant to levels seen with wild-type AE1. These oocyte data provide a mechanistic explanation for the normal anion transport function of G701D AE1 in erythrocytes containing GPA, but not in the distal kidney where α-intercalated cells lack GPA.

### Glyceraldehyde-3-phosphate dehydrogenase

Many glycolytic enzymes, including glyceraldehyde-3-phosphate dehydrogenase (GAPDH), aldolase, phosphofructokinase, lactate dehydrogenase, and pyruvate kinase bind to either AE1 itself or AE1-associated proteins (Chu and Low, 2006; Campanella et al., 2008). With the exception of GAPDH, the influence of glycolytic enzymes on AE1 activity is poorly understood. GAPDH catalyzes the conversion of glyceraldehyde-3-phosphate to D-glycerate-1,3-bisphosphate in the sixth step of glycolysis. GAPDH stimulated eAE1 expression in the basolateral membrane of MDCKI cells (Su et al., 2011). The C terminus of AE1 is responsible for the interaction. kAE1, but not a variant truncated at the C terminus by 11 residues, bound GAPDH in a yeast 2-hybrid screen (Su et al., 2011). GAPDH also interacts with eAE1 from rat erythrocytes and kAE1 from rat liver based on co-immunoprecipitation results (Su et al., 2011). GAPDH also binds to AE2 through a common anion exchanger C-terminal motif DEYxE (Su et al., 2011), although the functional consequences of this GADPH-AE2 interaction have not been demonstrated.

### Adaptor protein-1

Adaptor Protein-1 (AP-1) facilitates the exit of cargo proteins from the trans-Golgi by promoting the binding of clathrin. An siRNA-mediated decrease in the μ1A subunit of AP-1 inhibited the surface expression of co-transfected kAE1 in HEK293T cells (Sawasdee et al., 2010). In a similar study on MDCK1 cells, μ1A siRNA caused intracellular retention of human kAE1 (Almomani et al., 2012). Basolateral targeting of kAE1 was rescued by co-transfecting an siRNA-resistant μ1A subunit. In the same MDCK1 study, similar siRNA results were obtained for the μ1B subunit of AP-1. The AP-1 interaction with kAE1 requires the transporter's C terminus. The C-terminal 36 residues of kAE1 (bait) bound to the μ1A subunit of AP-1 in a yeast two-hybrid screen of a human kidney cDNA library (Sawasdee et al., 2010). AP-1 binding was confirmed and assigned to the kAE1 motif 904YDEV based on results from mutagenesis.

### Src-family kinases

Src-family kinases are tyrosine kinases encoded by the *Src* gene, and their phosphorylation of AE1 stimulates rapid internalization of the transporter (Yannoukakos et al., 1991; Williamson et al., 2008). In kAE1-expressing MDCK1 cells, kAE1 residues Y359 and Y904 were not phosphorylated under basal conditions (Williamson et al., 2008). However, treating the cells with the phosphatase inhibitor pervanadate caused phosphorylation of these two residues, and subsequent internalization of AE1. A variety of Src-family kinase inhibitors blocked this pervanadate effect. Curiously, the kAE1 point mutants Y359A and Y904A —both of which cannot be phosphorylated— displayed intracellular retention. These data are consistent with both the type of residue at position 359 and 904 and its phosphorylation state (by Src-family kinases) modulating plasma membrane expression of AE1. The authors proposed that a stimulus (e.g., a change in pH_i_) that shifts the balance of tyrosine kinase and phosphatase activities could alter kAE1 activity. This mechanism might be physiologically important, and responsible for basolateral AE1 stimulation in α-intercalated kidney cells that promotes transepithelial acid secretion during metabolic acidosis, and inhibition that reduces acid secretion during metabolic alkalosis.

### Protein 4.2

Protein 4.2 is an ATP-binding membrane protein found in erythrocytes. Protein 4.2 maintains stability and integrity of the erythrocyte membrane based on the observation that patients with protein 4.2 deficiencies present with spherocytosis and hemolytic anemia (Sung et al., 1992). In co-expression studies, protein 4.2 stimulated eAE1-mediated ^36^Cl^−^ uptake, and the proteins co-immunoprecipitated (Toye et al., 2005). Three mutant protein 4.2s that cause hereditary spherocytosis were also assayed for binding and function. Two of these proteins, the Tozeur and Komatsu protein 4.2 mutants, did not stimulate or bind eAE1, and also failed to traffic to the membrane (Toye et al., 2005). The other mutant (Nippon) did traffic to the membrane and had similar stimulatory effects on eAE1 as wild-type protein 4.2— a finding consistent with proper trafficking of protein 4.2 being required for full AE1 stimulation. The mechanism by which protein 4.2 stimulates eAE1 at the membrane is not clear, and may involve either an increase in AE1 expression or transporter activity.

### Protein 4.1

The N paralog of protein 4.1 is associated with the cytoskeleton in erythrocytes and provides structural rigidity to the membrane by stabilizing the spectrin-actin interaction (Baines et al., 2014). It is well established that the cytoplasmic domain of the erythrocyte AE1 protein binds protein 4.1 (Pasternack et al., 1985; Jöns and Drenckhahn, 1992). Protein 4.1 binds dimeric AE1 in forming a protein 4.1-GPC junctional complex, which also includes the proteins adducin, p55, and Rh (van den Akker et al., 2010). However, protein 4.1 regulation of AE1 is less established. Protein 4.1 may contribute to the confirmation of AE1, or regulate transport activity/expression in association with other regulatory proteins, e.g., through crosslinking (Baines et al., 2014). It is worth noting that the B paralog of protein 4.1 has been reported to bind NBCe1 in the kidney proximal tubule in association with p55 (Terada et al., 2007). Thus, protein 4.1 regulation of BTs may not be restricted to AE1. Protein 4.1 may also stimulate the activity of NCBTs by altering their conformation or attracting other regulatory proteins.

### Nephrin

Nephrin is a protein necessary for proper glomerular filtration, and is required for kAE1 expression in glomeruli. Native kAE1 expression is inhibited in human glomeruli with the nephrin mutation NPHS1(FinMaj) (Wu et al., 2010). Furthermore, transfecting wild-type nephrin into podocytes from these glomeruli restored kAE1 expression. According to results from a yeast two-hybrid screen of a human kidney cDNA library, a C-terminal region of AE1 (877–911) binds nephrin— a finding confirmed in immunoprecipitation studies with HEK293 cells heterologously expressing AE1 mutants. Mutual expression is further evident from the observation that nephrin expression is reduced in the AE1-knockout mouse (Wu et al., 2010).

**Figure 3:**
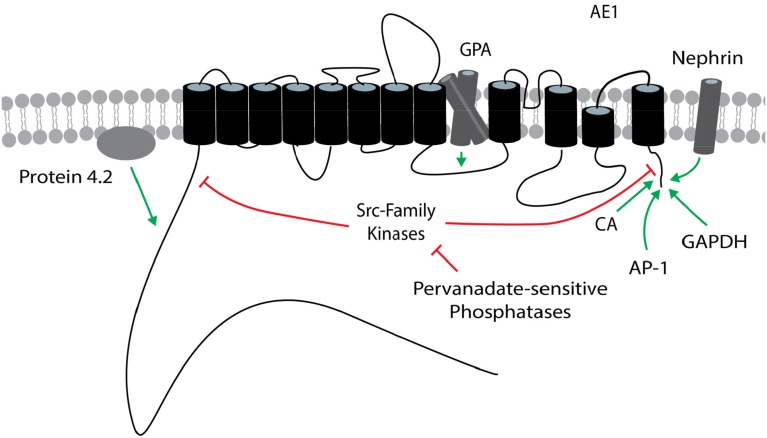
**Regulators of AE1**. The general position(s) of known or potential target site(s) within AE1 for each regulator is/are shown. Stimulatory effects are depicted by green arrows and inhibitory effects are depicted by red lines. These effects involve changes in transport function and/or plasma membrane expression, and may be variant dependent. The target site of protein 4.2 is unknown within the cytosolic N terminus of AE1. The AE1 topology is based on that presented in Cordat and Reithmeier (2014). AE1, anion exchanger 1; AP-1, adapter protein-1; CA, carbonic anhydrase; GAPDH, glyceraldehyde-3-phosphate dehydrogenase; GPA, glycophorin A.

Regulators of cloned AE1 variants are shown in Figure 3.

## NBCe1 (*Slc4a4*)

**Figure 4:**
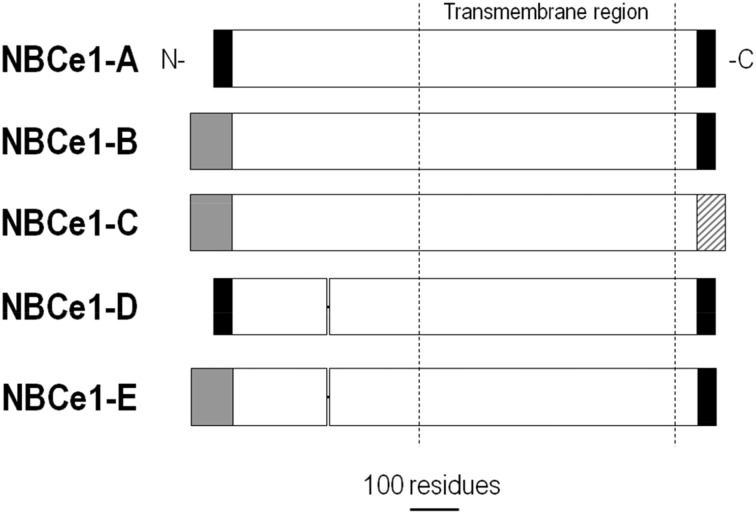
**Alignment of NBCe1 variants**. NBCe1 variants are identical except for differences at the N- and/or C termini, and/or the inclusion/exclusion of a 9-residue cassette within the cytoplasmic N-terminal region. Modified with permission from Thornell et al. (2012).

NBCe1 variants are found in many tissues where they are responsible for pH_i_ regulation, as well as solute absorption and secretion by epithelia. As depicted in Figure 4, NBCe1-A contains a different N terminus than NBCe1-B/C, and NBCe1-C contains a different C terminus than NBCe1-A/B. NBCe1-D is identical to NBCe1-A, except for the absence of the amino-acid sequence RMFSNPDNG found within the common N-terminal region of NBCe1-A/B/C (i.e., after the variable N-terminal region and before the transmembrane region in Figure 4). NBCe1-E is identical to NBCe1-B, but lacks the aforementioned RMFSNPDNG amino acid sequence. The function of NBCe1-D and -E remains to be examined. NBCe1-A/B/C —and presumably the D and E variants— have either a 1:2 or 1:3 Na^+^:HCO^−^_3_ stoichiometry.

NBCe1-A plays a major role in HCO^−^_3_ reabsorption in the proximal tubule of the kidney. Filtered HCO^−^_3_ and secreted H^+^ into the tubule lumen are converted to CO_2_ and H_2_O in a net reaction catalyzed by luminal CAIV activity. Generated CO_2_ in the tubule lumen diffuses into the epithelial cell where intracellular CAII converts the CO_2_ plus H_2_O back into H^+^ and HCO^−^_3_. The H^+^ is secreted into the tubule lumen for subsequent reactions with more HCO^−^_3_. The intracellular HCO^−^_3_ is reabsorbed into the blood via a basolateral NBCe1-A (Boron and Boulpaep, 1983) with a 1:3 stoichiometry (Soleimani et al., 1987).

NBCe1-B is also located in the basolateral membrane of epithelial cells, and has been shown to contribute to HCO^−^_3_ secretion in many tissues, including the pancreas (Muallem and Loessberg, 1990) and airway (Garnett et al., 2011; Shan et al., 2012). In these epithelia, the inward transport of net-negative charge and HCO^−^_3_ helps establish the electrical gradient and HCO^−^_3_ chemical gradient that drive HCO^−^_3_ secretion across the apical membrane. The electrical gradient can also drive the apical secretion of additional anions, particularly Cl^−^. In addition to transporting solutes across epithelia, NBCe1-B/C contribute to general pH_i_ shifts, which are especially significant in the nervous system where corresponding changes in pH_o_ can modulate neuronal firing (Chesler, 2003). The transporter in glial cells such as astrocytes plays a particularly important role in coupling pH changes with voltage changes. More specifically, following neuronal firing, elevated extracellular K^+^ depolarizes the membrane of astrocytes, thereby stimulating the electrogenic NBCe1. This NBCe1-mediated HCO^−^_3_ transport alkalinizes the astrocyte, as well as acidifies the extracellular space that tends to dampen further neuronal activity. Neurons also express NCBTs, particularly electroneutral ones such as NBCn1 and NDCBE (Majumdar and Bevensee, 2010). Thus, NBCe1 is likely the dominant NCBT responsible for the pH_i_ physiology of astrocytes and activity-evoked pH_o_ changes, whereas neuronal electroneutral NCBTs contribute to the pH_i_ physiology of neurons independent of voltage changes and therefore make a smaller contribution to activity-evoked pH_o_ changes. However, this profile is likely an oversimplification based on the more-recent evidence that neurons can also exhibit functional electrogenic NBC activity (Majumdar, 2009; Majumdar and Bevensee, 2010; Svichar et al., 2011).

### Carbonic anhydrase

Carbonic anhydrase (CA) catalyzes the slow reaction H^+^ + HCO^−^_3_ ↔ CO_2_ + H_2_O. While monitoring NBCe1 short-circuit current, Gross et al. (2002) found that the CA inhibitor acetazolamide reduced the activity of NBCe1-A operating with a 1:3, but not a 1:2 Na^+^:HCO^−^_3_ stoichiometry. The authors proposed that phosphorylation of S982, which is responsible for the stoichiometry change to 1:2 (Gross et al., 2002), eliminates CA binding to NBCe1-A. Interestingly, S982 lies in close proximity to a predicted CA binding site 986DNND, which is similar to the CAII-binding sequence DADD of AE1 (Vince and Reithmeier, 1998).

The Kurtz group proposed that 958LDDV was another CAII-binding site in NBCe1-A (Pushkin et al., 2004). Pushkin et al. (2004) used pull-down assays and measured acetazolamide-sensitive currents for NBCe1 constructs expressed in a mouse proximal tubule cell line, where heterologously expressed NBCe1-A displays a 1:3 Na^+^:HCO^−^_3_ stoichiometry (Gross et al., 2001a,b). The authors found that the degree of acetazolamide inhibition of NBCe1 correlated with the degree of CAII/NBCe1-A binding. NBCe1-A mutants that bound CAII weakly were more insensitive to acetazolamide than wild-type NBCe1-A, which displayed the strongest CAII binding. In the proposed model, 958LDDV and 986DNND are part of a single binding site. Indeed, according to results from isothermal titration calorimetry, human kidney NBCe1-A has 1 high-affinity binding site for CAII with a binding constant of 160 nM (in protein-free PBS buffer) (Gross et al., 2002). CAII activity also appears to stimulate other NBCe1 variants. Expressing a catalytically reduced CAII mutant in HEK293 cells reduced the activity of co-expressed NBCe1-B (Alvarez et al., 2003).

Similar to CAII regulation of AE1, direct binding of CAII to NBCe1 remains controversial. In the aforementioned CAII/NBCe1 studies, reducing CAII activity or mutating the putative CAII binding site on NBCe1 inhibited NBCe1 activity. However, injecting *Xenopus* oocytes with CAII protein failed to stimulate NBCe1-A (Lu et al., 2006). CAII was functional in these experiments based on the observation that pH_i_ recoveries from CO_2_-induced acidifications were faster and were inhibited by the membrane-permeant CAII inhibitor ethoxzolamide. In additional experiments, the authors found that CAII fused to a GFP-tagged NBCe1-A construct (eGFP-NBCe1-A) displayed no enhanced NBC activity over the control eGFP-NBCe1-A construct. Furthermore, no co-localization of NBCe1-A and endogenous CAII was evident compared to that seen with CAII synthetically conjugated to NBCe1-A. Discrepancies in these findings from those of the Pushkin et al. study may reflect different cell type-dependent NBCe1 stoichiometries. NBCe1-A has a 1:2 Na^+^:HCO^−^_3_ when expressed in *Xenopus* oocytes (Heyer et al., 1999; Sciortino and Romero, 1999; McAlear et al., 2006), but a 1:3 Na^+^:HCO^−^_3_ stoichiometry when expressed in the proximal tubule cell line used in the Pushkin et al. study (Gross et al., 2001a).

In a more recent *Xenopus* oocyte study, Schueler et al. (2011) found that CAI-CAIII all stimulated NBCe1-A-mediated pH_i_ recoveries and currents when oocytes were held at −40 mV. CAI lacks the putative N-terminal residues for NBCe1-A binding (3HH… 9KH… 15H… 17H), and CAIII has alternative residues 3E and 9S within this domain (Schueler et al., 2011). Thus, these experiments support the contention that an interaction is not necessarily required for CA stimulation of NBCe1.

CAIV, an outer plasma membrane glycosylphosphatidylinositol-anchored CA, also stimulated NBCe1-B co-expressed in HEK293 cells (Alvarez et al., 2003). The authors also showed by pull-down assays and immunodetection that CAIV —obtained by lysing CAIV-expressing cells— bound to GST-conjugated NBCe1-B. CAIV binding to and functional stimulation of NBCe1-B was dependent on residue G767 found in the fourth extracellular loop of NBCe1-B that is conserved in CAIV-binding AE1, but not in AE3 (Alvarez et al., 2003). CAIV is expected to stimulate all NBCe1 variants, which have this conserved glycine at position 767.

### Autoregulation

When expressed in *Xenopus* oocytes, NBCe1-A has ~four-fold greater activity than NBCe1-B or -C (McAlear et al., 2006). NBCe1-A differs from NBCe1-B and -C at its N terminus (Figure 4), and removing this different N terminus reduces NBCe1-A activity by approximately half. Thus, there appears to be an autostimulatory domain (ASD) within this N terminus of the A variant that stimulates activity. The ASD is also expected to contribute to the activity of NBCe1-D, which has the same N terminus (Figure 4). Currently, there are no known regulators of the ASD. This ASD may contribute to the efficient reabsorption of HCO^−^_3_ in the kidney by promoting high-velocity NBCe1-A-mediated transport of HCO^−^_3_ across the basolateral membrane in the proximal tubule.

Autoregulation has also been demonstrated for the N terminus of NBCe1-B and -C. Truncating the N terminus of NBCe1-C stimulated the transporter 2.7-fold (McAlear et al., 2006). Furthermore, the activity of this truncated variant was similar to that of the corresponding NBCe1-A truncation (i.e., they had similar whole oocyte currents). Therefore, the N terminus of NBCe1-B or -C appears to contain an autoinhibitory domain (AID). As described below, this AID is at least partially regulated by other binding proteins, especially inositol 1,4,5-trisphosphate (IP3) receptor binding protein released with IP3 (IRBIT). NBCe1-E is also predicted to have an AID because it contains the same N terminus as NBCe1-B and -C (Figure 4).

The specific C terminus of NBCe1-C may also influence regulation by the AID based on the following two observations with NBCe1 stimulators soon to be discussed. First, co-expressing IRBIT in oocytes stimulated co-expressed NBCe1-C to a greater extent (i.e., more relief of the AID) than NBCe1-B (Thornell et al., 2010). Second, removing the AID-containing N terminus of NBCe1-C —but not NBCe1-B— resulted in a small but significant IP_3_-induced stimulation that required ER-store Ca^2+^ (Thornell et al., 2012). Based on these observations, the C terminus of NBCe1-C either contributes to a joint N-terminal/C-terminal AID or contains a separate AID.

### IRBIT-WNK/SPAK

IRBIT is a ubiquitous second messenger protein that binds to the IP_3_ receptor and competes with intracellular IP_3_ (Ando et al., 2003). IRBIT released from the IP_3_ receptor into the cytosol regulates many proteins, including channels and transporters (Ando et al., 2014). When heterologously expressed in *Xenopus* oocytes, IRBIT stimulated human NBCe1-B, but not NBCe1-A (Shirakabe et al., 2006). In similar oocyte co-expression experiments, IRBIT also stimulated rat NBCe1-B and -C, but not mutants with N-terminal truncations (Thornell et al., 2010). In co-immunoprecipitation studies, IRBIT bound to NBCe1-B, but not NBCe1-A (Shirakabe et al., 2006)— data consistent with IRBIT binding to the different N terminus of the B and C variants. Mutating the phosphorylated residue S68 or S71 in IRBIT's PEST domain to an Ala still enabled IRBIT to bind to the IP_3_ receptor (Ando et al., 2009). However, these mutants failed to bind to and stimulate NBCe1-B (Shirakabe et al., 2006), and failed to stimulate NBCe1-C (Thornell et al., 2012). Thus, there are different regions of IRBIT responsible for IP_3_ receptor binding and NBCe1 stimulation.

Results from pull-down assays comparing the binding of IRBIT to different serial truncations of NBCe1-B indicated that amino acid regions 1–18 and 37–62 were necessary for IRBIT binding (Shirakabe et al., 2006). In expression studies with HeLa cells, Hong et al. (2013) demonstrated that a stretch of arginines (42–44) within the positively charged region 37–65 of NBCe1-B is required for IRBIT binding and stimulation. Double, triple, but not single, arginine to alanine replacements greatly reduced IRBIT binding, while the triple replacement eliminated IRBIT stimulation of transport. In addition to these basic arginine residues, T49 is also required for IRBIT regulation. In a separate study, NBCe1-B amino acid residues between 4 and 16 were also required for IRBIT stimulation (Lee et al., 2012c). These functional data support Shirakabe et al.'s prediction that the IRBIT binding and stimulatory determinants of NBCe1-B involve multiple motifs within a complex tertiary structure rather than a single motif (Shirakabe et al., 2006).

Seki et al. (2008) proposed that IRBIT stimulates NBCe1-B by removing the AID inhibition. If true, then expressing IRBIT and removing the AID should stimulate NBCe1 activity to the same extent. IRBIT contains a binding domain for protein phosphatase-1 (PP-1) (Devogelaere et al., 2007). As such, it is difficult to activate IRBIT fully because it is normally inhibited by the ubiquitous phosphatase PP-1. To activate IRBIT maximally, the Boron group mutated IRBIT's PP-1 binding site to create a super-IRBIT (Lee et al., 2012c). The activity of NBCe1-B co-expressed with super-IRBIT exceeded the activity of the N-terminal truncation of NBCe1-B lacking the putative N-terminal AID (Lee et al., 2012c). The authors concluded that IRBIT did not stimulate NBCe1-B through relief of the AID exclusively. The AID's tertiary structure may include other parts of the transporter, such as a putative C-terminal AID as mentioned above.

**Figure 5:**
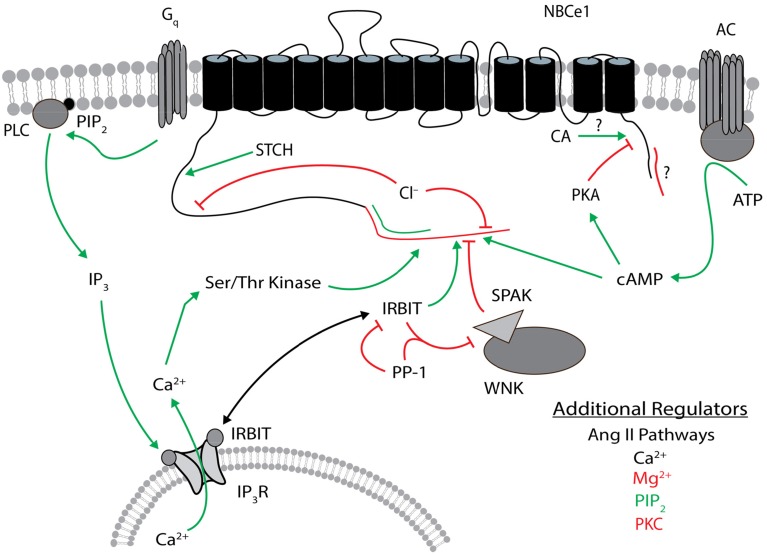
**Regulators of NBCe1**. The general position(s) of known or approximate target site(s) within NBCe1 for each regulator is/are shown. Stimulatory effects are depicted by green arrows and inhibitory effects are depicted by red lines. The N terminus of NBCe1-B and -C that contains the autoinhibitory domain is shown in red, and the different N terminus in -A that contains the autostimulatory domain is shown in green. The different C terminus of NBCe1-C (vs. -A/B) that contains a possible autoinhibitory domain is shown in red. Direct binding of carbonic anhydrase to the C terminus remains controversial. The target site of STCH is unknown within the cytosolic N-terminal residues 96–440 of NBCe1-B. Ser/Thr kinase involves a site within the different N terminus of the B/C variants. Additional regulators listed are not as well characterized, especially regarding their target sites on NBCe1. These additional regulators in black type can be either stimulatory or inhibitory. Regulatory effects involve changes in transport function and/or plasma membrane expression, and may be variant dependent. The NBCe1 topology is based on that presented in Parker and Boron (2013). CA, carbonic anhydrase; IP_3_, inositol 1,4,5-trisphosphate; IP_3_R, IP_3_ receptor; IRBIT, IP_3_R binding protein released with IP_3_; PIP_2_, phosphatidylinositol 4,5-bisphosphate; PKA, protein kinase A, PKC, protein kinase C; PLC, phospholipase C; PP-1, protein phosphatase 1; SPAK, STE20/SPS1-related proline/alanine-rich kinase; STCH, Hsp70-like stress 70 protein chaperone; WNK, with-no-lysine kinase.

IRBIT and its binding partner PP-1 are linked to other regulatory molecules (Figure 5). PP-1 antagonizes the WNK (with-no-lysine kinase)/SPAK (STE20/SPS1-related proline/alanine-rich kinase) pathway (Yang et al., 2011). Human WNK/SPAK mutations cause hypertension— a finding that implicates this pathway as a regulator of epithelial electrolyte transport (Wilson et al., 2001). Indeed, WNK/SPAK regulates many epithelial ion channels and transporters including the Na/K/2Cl cotransporter (Anselmo et al., 2006), the epithelial sodium channel (Heise et al., 2010), the cystic fibrosis transmembrane conductance regulator (Yang et al., 2007), the renal outer medullary K^+^ channel (He et al., 2007), and NBCe1-B and -C (Yang et al., 2009).

In HeLa cells, the WNK/SPAK pathway also inhibits NBCe1-B (Yang et al., 2011; Hong et al., 2013). Transfecting either WNK or kinase-dead WNK mutants inhibited NBCe1-B— consistent with WNK acting as a scaffold, not a kinase, for another NBCe1 regulator such as SPAK (Yang et al., 2011). Co-transfecting kinase-dead SPAK with WNK blocked the WNK-mediated inhibition of NBCe1. The phosphorylation of S65 by SPAK was required for the SPAK-mediated decrease in NBCe1-B surface expression (Hong et al., 2013). These data are consistent with WNK recruiting SPAK to phosphorylate NBCe1-B and to reduce NBCe1-B surface expression. This WNK/SPAK-mediated NBCe1-B inhibition was antagonized by IRBIT recruitment of the phosphatase PP-1 (Yang et al., 2011; Hong et al., 2013). The phosphorylation of NBCe1-B residue T49 is also required for both the IRBIT and WNK/SPAK pathways, and the T49A substitution eliminated regulation by both proteins (Hong et al., 2013). The WNK/SPAK and IRBIT/PP-1 pathways have also been reported to converge with the regulator PIP_2_, which also appears to require the positively charged N terminal region 37–65 (Hong et al., 2013). However, the IP_3_/Ca^2+^ pathway may be involved. In summary, whereas IRBIT stimulates NBCe1-B and -C, WNK/SPAK antagonizes these NBCe1 variants (see review by Park et al., 2012). In addition to the direct IRBIT stimulation of NBCe1, IRBIT indirectly disinhibits NBCe1 from WNK/SPAK by recruitment of PP-1. This signaling complex likely regulates fluid and electrolyte transport in the pancreatic duct (Yang et al., 2009, 2011; Park et al., 2012). For example, purinergic hormones and cholecystokinin that stimulate pancreatic HCO^−^_3_ secretion through an increase in intracellular IP_3_ are expected to displace IRBIT from the IP_3_ receptor. This cytosolic IRBIT now inhibits WNK/SPAK and increases basolateral NBCe1 activity (responsible for HCO^−^_3_ uptake from the interstitium), as well as apical CFTR and Slc26a6 activities (both responsible for HCO^−^_3_ transport into the duct). We recommend Lee et al. (2012a) for a more detailed description of pancreatic HCO^−^_3_ secretion.

IRBIT's effect on NBCe1-B surface expression depends on the expression system. Co-expressing IRBIT and NBCe1-B in *Xenopus* oocytes did not change surface NBCe1 protein assayed by confocal imaging (Shirakabe et al., 2006), single-oocyte chemiluminescence (Thornell et al., 2010), or biotinylation and subsequent immune-detection (Lee et al., 2012c). However, IRBIT increased NBCe1-B surface expression in HeLa cells (Yang et al., 2011). The basis of this discrepancy may be a less active WNK/SPAK pathway in oocytes than HeLa cells. If so, then the majority of NBCe1-B would already be expressed on the oocyte membrane, and only an expression-independent increase in transporter current is observed. Consistent with this explanation, co-expressing NBCe1-B and IRBIT in HeLa cells suppressed WNK/SPAK-mediated NBCe1 internalization and stimulated NBCe1-B by a WNK/SPAK-independent mechanism (Hong et al., 2013)— most likely by the expression-independent mechanism characterized for IRBIT and NBCe1 in oocytes.

### PIP_2_

Phosphatidylinositol 4,5-bisphosphate (PIP_2_) is a minor membrane phospholipid involved in many cellular processes including the regulation of channels and transporters (Di Paolo and De Camilli, 2006; Balla, 2013). The rat-kidney NBCe1-A current displays rundown in a macropatch of *Xenopus* oocyte membrane that can be inhibited and reversed by directly applying a fast membrane incorporating short-chain PIP_2_ (Wu et al., 2009). In addition, NBCe1-A current rundown is inhibited by vanadate and Mg^2+^-free solutions. These data are consistent with an initial, endogenous PIP_2_-dependent transporter current that rapidly decays due to Mg^2+^-sensitive phosphatase activity that decreases PIP_2_.

These findings in isolated membrane patches have been extended to the whole oocyte. Injecting exogenous PIP_2_ into an oocyte to raise PIP_2_ is not straightforward because the injected PIP_2_ is rapidly hydrolyzed to IP_3_ by phospholipase C (PLC) (Thornell et al., 2012). Nonetheless, blocking PIP_2_ hydrolysis by pretreating the oocyte with the PLC inhibitor U73122 resulted in a modest PIP_2_ injection-induced stimulation of NBCe1-A, -B, and -C (Thornell et al., 2012). Importantly, NBCe1-A was not stimulated by the PIP_2_ injection in the absence of U73122. Therefore, the PIP_2_-induced stimulation observed in the presence of U73122 was not the result of incomplete PLC inhibition. The modest PIP_2_-induced stimulation of NBCe1 may indicate that PIP_2_ is near it maximal effective concentration in oocytes. As such, decreasing PIP_2_ may be a better assay for exploring PIP_2_ regulation of NBCe1.

We have recently found that a decrease in membrane PIP_2_ itself —independent of IP_3_— inhibits NBCe1-B and -C expressed in *Xenopus* oocytes (Thornell and Bevensee, 2015). Degrading PIP_2_ was achieved by co-expressing NBCe1 with a voltage-sensitive phosphatase (VSP) that dephosphorylates PI(4,5)P_2_ to PI(4)P. Activating VSP by sufficient depolarization inhibited NBCe1-B and -C. Importantly, depolarizing oocytes to voltages that do not sufficiently activate the VSP, or using a phosphatase-dead mutant (C366S VSP) did not inhibit NBCe1-mediated currents or pH_i_ recoveries from an acid load. Furthermore, in simultaneous voltage-clamp and confocal imaging experiments with a PIP_2_-sensitive fluorescent probe, VSP-elicited changes in NBCe1 current mirrored PIP_2_ changes in the plasma membrane.

Where might PIP_2_ bind to NBCe1? Based on crystallography data of the Kir2.2-PIP_2_ and the GIRK2-PIP_2_ interactions (Hansen et al., 2011; Whorton and MacKinnon, 2011), these K^+^ channels have a non-specific phospholipid domain within the transmembrane (TM) region, as well as a polycationic PIP_2_ binding domain in the cytosol near one TM region. NBCe1 variants contain similar PIP_2_-binding motifs, including KDKKKKEDEKKKKKKK in the cytosolic C-terminus, RKHRH in the cytosolic N terminus, and RKEHKLKK before TM8. The region before TM8 is of particular interest because TM8 is involved in ion translocation (McAlear and Bevensee, 2006). An additional PIP_2_-binding motif, RRRRRHKRK is found in the N terminus of NBCe1-B and -C. Recently, Hong et al. (2013) reported that a trimer of arginines (42–44) within this region is required for PIP_2_ stimulation of NBCe1-B. However, this region may play a role in IP_3_/Ca^2+^ regulation instead. This region is unlikely to be the sole PIP_2_-binding region because it is absent in the PIP_2_-sensitive NBCe1-A.

### IP_3_ and Ca^2+^

Injecting PIP_2_ into whole *Xenopus* oocytes expressing NBCe1-B or -C, but not NBCe1-A, stimulated the NBC current (Thornell et al., 2012). The majority of this stimulation was mediated by PIP_2_ hydrolysis to IP_3_, required an intracellular Ca^2+^ store, and involved a staurosporine-sensitive Ser/Thr kinase (Thornell et al., 2012). Raising intracellular Ca^2+^ (e.g., by applying ionomycin and activating membrane Ca^2+^ channels and G_q_-coupled receptors) also stimulated the B and C variants. This mode of stimulation also required the distinct N terminus of the B/C variant. Thus, kinase-mediated phosphorylation of NBCe1 or an NBCe1 regulator may relieve the N-terminal AID. As previously reported, such a kinase pathway may also be responsible for observed ATP stimulation of NBCe1-A in the macropatch (Heyer et al., 1999). Activating endogenous G_q_-coupled receptors in *Xenopus* oocytes by applying lysophosphatidic acid (LPA) stimulated the activity of heterologously expressed NBCe1-C. However, LPA stimulated the activity of heterologously expressed NBCe1-B by an increase in expression in physiological conditions (Thornell et al., 2012). In recent experiments, LPA stimulated NHE3 expression through the Ser/Thr kinase p90 ribosomal S6 kinase (RSK) (No et al., 2015). These similarities raise the possibility that RSK may be involved in LPA-induced stimulation of at least NBCe1-B.

NBCe1-A expressed in whole *Xenopus* oocytes was minimally stimulated by PIP_2_ injection, and unaffected by raising intracellular Ca^2+^, e.g., by activating store-operated Ca^2+^ channels (Thornell et al., 2012). However, in a separate study, raising bath Ca^2+^ from 100 to 500 nM stimulated the slope conductance of rat NBCe1-A in a fraction of inside-out oocyte macropatches. Based on I-V data, the stimulation involved an increase in the stoichiometry from 1:2 to 1:3 Na^+^:HCO^−^_3_(Müller-Berger et al., 2001). Thus, Ca^2+^ regulation of NBCe1-A is clearly different in the whole cell vs. at least a fraction of isolated membrane patches.

#### PIP_2_ vs. IP_3_/Ca^2+^

The aforementioned evidence that PIP_2_ can stimulate all three NBCe1 variants directly, or NBCe1-B and -C indirectly through its hydrolysis and subsequent IP_3_/Ca^2+^, raises the question of which is the dominant pathway (Thornell and Bevensee, 2015). This dual mode of regulation is similar to that reported for the KCNQ-mediated M current in superior cervical ganglion cells (Zhang et al., 2003; Gamper et al., 2004; Suh and Hille, 2005; Winks et al., 2005; Zaika et al., 2011). We believe the inhibitory effect of PIP_2_ hydrolysis will dominate when G_q_ receptors are activated away from a Ca^2+^ source. Thus, there is no Ca^2+^ release that can activate lipid kinases that normally replenish the PIP_2_. In contrast, the stimulatory effect of PIP_2_ hydrolysis to IP_3_/Ca^2+^ will dominate when the receptors are activated near a Ca^2+^ source. Alternatively, the IP_3_/Ca^2+^ pathway may predominate under physiological conditions with adequate energy levels. However, the PIP_2_ pathway would play the dominant role with energy (ATP) depletion, for example, associated with anoxia or hypoxia. An associated decrease in PIP_2_ may inhibit transporters such as NBCe1 in a protective way to minimize large intracellular Na^+^ and Ca^2+^ loads and/or to conserve remaining energy. Such inhibition may counteract the deleterious effect of NBCe1 stimulation in the ischemic heart as described above. Finally, as suggested by Hilgemann et al. (2001), the PIP_2_ requirement for NBCe1 function may keep the transporters necessarily inactive during maturation and trafficking through organelles prior to expression at the plasma membrane.

### Angiotensin II, PKC, and Ca^2+^

It is well established that endogenous NBCe1-A is stimulated by low concentrations (10^−11^–10^−9^ M) of angiotensin II (Ang II) and inhibited by high concentrations (10^−9^–10^−6^ M) of Ang II (Geibel et al., 1990; Eiam-Ong et al., 1993; Coppola and Frömter, 1994; Ruiz et al., 1995; Robey et al., 2002; Zheng et al., 2003). Ang II-mediated bimodal regulation has been reported for NBCe1-A heterologously expressed in *Xenopus* oocytes co-expressing the Ang II receptor AT_1A_ (Perry et al., 2006, 2007). Low concentrations of Ang II increased the depolarization-induced NBCe1 current through a Ca^2+^-dependent mechanism that could be inhibited by BAPTA (Perry et al., 2006). However, high concentrations of Ang II inhibited this NBCe1 current through a pathway involving Ca^2+^-insensitive protein kinase C, PKCε that reduced expression at the plasma membrane (Perry et al., 2006, 2007). Interestingly, both a PKCε inhibitor and the Ca^2+^ chelator BAPTA reduced the high Ang II-mediated NBCe1 inhibition, perhaps through separate pathways. Furthermore, the Ang II-induced inhibition was irreversible after applying calmodulin inhibitors or monensin— a finding consistent with high Ang II inhibiting NBCe1-A trafficking to the plasma membrane in oocytes. Thus, results from heterologous expression studies have provided mechanistic insight into the concentration-dependent, Ang II-mediated bimodal regulation of endogenous NBCe1 in kidney.

### Acetylcholine

Acetylcholine (ACh) is a neurotransmitter that activates either ionotropic or metabotropic receptors. Metabotropic ACh receptor activation promotes anion secretion in many epithelial cell types such as acinar cells in the pancreas (Steward and Ishiguro, 2009; Lee et al., 2012a). Curiously though, applying carbachol lowered membrane expression of both transiently expressed NBCe1-A and NBCe1-B in acinar ParC5 cells (Perry et al., 2009). Furthermore, contradictory to secretion observed in native tissue, pretreating NBCe1-B-expressing HEK293 cells with carbachol did not alter NBCe1-B activity (Bachmann et al., 2008). The paradoxical findings may be the result of NBCe1 overexpression or cell-type specific cAMP-mediated pathways, which can merge with metabotropic ACh signaling and jointly regulate anion secretion (e.g., in the pancreas, Lee et al., 2012a).

### cAMP

cAMP is a second messenger formed by adenylate cyclase (AC)-mediated catalysis of ATP. It is well established that a hormone-induced increase in cAMP decreases renal tubular HCO^−^_3_ absorption and increases pancreatic duct HCO^−^_3_ secretion (McKinney and Myers, 1980; Liu and Cogan, 1989; Ishiguro et al., 1996a,b; Kunimi et al., 2000). However, less is known about the molecular mechanisms by which cAMP alters the activity of NBCe1— the dominant Na^+^-coupled HCO^−^_3_ transporter in these tissues.

There is evidence that cAMP can contribute to a change in transport stoichiometry of NBCe1 from 1:3 to 1:2 Na^+^:HCO^−^_3_ through protein kinase A (PKA)-mediated phosphorylation of either S982 of the A variant or the homologous S1026 of the B variant (Gross et al., 2001a,b, 2003). Furthermore, the base stoichiometry and response to cAMP of NBCe1-A and -B have been reported to be cell-type dependent (Gross et al., 2003). In the kidney proximal tubule, this stoichiometry change would account for observed inhibition of net HCO^−^_3_ efflux by cAMP-elevating dopamine (Kunimi et al., 2000) and parathyroid hormone (McKinney and Myers, 1980). However, it is now clear that the regulation of NBCe1 stoichiometry is complex and not fully understood. For example, additional factors can influence NBCe1 stoichiometry, including intracellular Ca^2+^ (see above, Müller-Berger et al., 2001), other residues such as T485 (A variant), which is part of putative TM3 (Zhu et al., 2013), and even assay-dependent measurements (Zhu et al., 2013).

cAMP can also stimulate NBCe1-B activity, as indicated by an increase in slope conductance, without an apparent change in stoichiometry (Gross et al., 2003). cAMP stimulation of NBCe1-B required residue T49 located in the PKA phosphorylation site of the different N-terminus, although did not require a change in its phosphorylation state. Curiously, this Thr at position 49 in human NBCe1-B is not conserved across species, and is an Ala in rat NBCe1-B. This mode of cAMP stimulation was also observed for NBCe1-B expressed in mPT cells as indicated by an increase in slope conductance (in addition to the reversal potential shift described above). Secretin-induced HCO^−^_3_ secretion by the pancreatic duct may be due, at least in part, to cAMP stimulation of NBCe1-B in the basolateral membrane (Ishiguro et al., 1996a,b).

### Magnesium

Free cytosolic Mg^2+^ remains fairly constant in a variety of physiological conditions— ranging from 0.5 to 0.7 mM (Romani, 2007). Yamaguchi and Ishikawa (2008) expressed bovine NBCe1-B in HEK293 and NIH3T3 cells, and monitored whole-cell Na^+^-dependent HCO^−^_3_ currents by patch clamping while varying the Mg^2+^ concentration in the pipette solution. For HEK293 cells, Mg^2+^ inhibited Na^+^-dependent HCO^−^_3_ currents with K_i_ values that appeared voltage-independent (e.g., ~15 μM at −20 mV, ~11 μM at +20 mV, and ~11 μM at +40 mV). A similar Mg^2+^-induced NBCe1-B inhibition was observed for NIH3T3 cells. The AID of NBCe1-B was involved because an N-terminal truncation of NBCe1-B raised the K_i_ for Mg^2+^ to 300 μM.

Endogenous NBCe1-B in bovine parotid acinar cells was also inhibited by Mg^2+^ with ~eight-fold higher K_i_ values (Yamaguchi and Ishikawa, 2008). The rightward shift in K_i_ from the previous expression systems was likely due to cell-type specific regulation. For example, IRBIT co-transfection increased the K_i_ for Mg^2+^-induced inhibition of NBCe1-B stably transfected into HEK293 cells from 560 nM to 11 μM (Yamaguchi and Ishikawa, 2012). However, it is not clear why the K_i_ for Mg^2+^-induced NBCe1-B inhibition in this study was lower than in the previous study (Yamaguchi and Ishikawa, 2008).

Mg^2+^ may inhibit NBCe1-B by disrupting PIP_2_ stimulation of the transporter. More specifically, the cationic Mg^2+^ may screen the negative charges of PIP_2_ (Suh and Hille, 2007), which we have recently shown to stimulate NBCe1 (Thornell et al., 2012; Thornell and Bevensee, 2015). Consistent with this mechanism, other polyvalent cations such as neomycin (Yamaguchi and Ishikawa, 2008) and spermine (Yamaguchi and Ishikawa, 2012) increased the K_i_ for Mg^2+^-induced inhibition of NBCe1-B heterologously expressed in HEK293 and NIH3T3 cells, and endogenously expressed in bovine parotid acinar cells. Such polycations also inhibit PIP_2_ stimulation of NBCe1-A (Wu et al., 2009).

Although it is well established that Mg^2+^ interacts and regulates ion channels and transporters, including the Na-K and Ca^2+^ pumps Jorgensen et al., 2003; Lu, 2004; Romani, 2007, it is intriguing that Mg^2+^ also regulates NBCe1, and perhaps other NCBTs. Such regulation by divalents other than Ca^2+^ may play a much larger role than previously thought in acid-base handling in organs such as the kidney. As described above, cytosolic Mg^2+^ is relatively stable under physiological conditions. However, it can rise to over 1.0 mM under pathological conditions such as ischemia (Murphy et al., 1989). Perhaps elevated Mg^2+^-induced inhibition of NBCe1 protects cells in pathological states by minimizing large intracellular Na^+^ and Ca^2+^ loads— similar to that proposed above for PIP_2_ hydrolysis-induced inhibition of NBCe1.

### Chloride

In a recent study, Shcheynikov et al. (2015) reported that intracellular Cl^−^ (Cl^−^_i_) is a signaling ion that regulates NBCe1-A and -B, as well as NBCe2-C expressed in HeLa cells. High Cl^−^_i_ inhibits NBCe1-B and involves two GxxxP sites in the cytosolic N terminus. One is a low-affinity site active under basal conditions, whereas the other is a high-affinity site unmasked by IRBIT. Interestingly, NBCe1-A is insensitive to Cl^−^_i_ in the physiological range, but does contain a single, low-affinity GxxxP site in its N terminus that can be unmasked by deleting residues 29–41. NBCe2-C has a single, high-affinity GxxP in its cytosolic N terminus that is basally active. Cl^−^_i_ regulation of NBCe1 appears to be cell-type dependent because IRBIT stimulates NBCe1-B expressed in oocytes, which have a high Cl^−^_i_ [~35 mM (Cooper and Fong, 2003)]. This oocyte Cl^−^ concentration inhibits the transporter in HeLa cells under the same conditions. As the authors propose, Cl^−^_i_ regulation of NBCe1-B could serve to couple NBCe1 and AE activity in a negative feedback fashion, and to promote continued HCO^−^_3_ secretion by epithelia in distal portions of ducts (e.g., salivary and pancreatic) where Cl^−^_i_ is low.

### Chaperone stress 70 protein (STCH)

Chaperone stress 70 protein (STCH) is a ubiquitously expressed microsomal-associated protein whose cellular function is poorly understood (Bae et al., 2013). Although a member of the 70-kDa heat-shock protein family, STCH is not activated by heat shock. STCH is expressed ubiquitously and activated by Ca^2+^. STCH binds to NBCe1-B based on results from a yeast 2-hybrid screen (Bae et al., 2013). Co-expressing STCH with NBCe1-B in *Xenopus* oocytes caused an increase in transporter current due to enhanced protein expression. The N terminus of NBCe1 was not involved because STCH co-expression also stimulated an NBCe1 truncated at its N terminus (Δ95aa). Furthermore, STCH and IRBIT stimulation of NBCe1-B was additive in co-expression studies, and therefore likely involve separate pathways. STCH stimulation of endogenous NBCe1-B was evident in a human submandibular gland ductile cell line, HSG (Bae et al., 2013). siRNA knockdown of STCH in HSG cells inhibited a DIDS-sensitive pH_i_ recovery from an NH^+^_4_-induced acid-load, and decreased membrane expression of NBCe1-B. Bae et al. (2013) noted that STCH is down regulated in some cancers and central nervous system disorders, and the reduced stimulation of NBCe1 would promote cellular acidification.

Regulators of cloned NBCe1 variants are shown in Figure 5.

## NBCn1 (*Slc4a7*)

**Figure 6:**
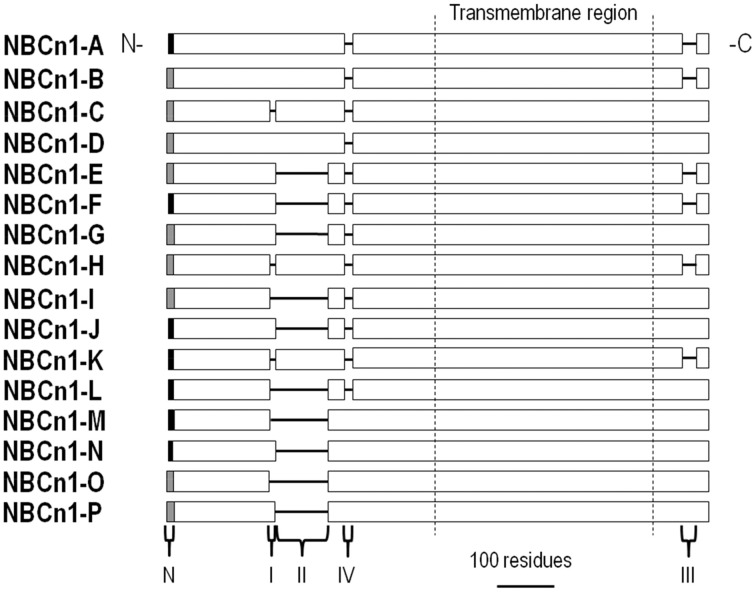
**Alignment of NBCn1 variants**. NBCn1 variants are identical except for a mixture of different variable N termini (N), a 13-residue cassette I in the cytoplasmic N-terminal region, a 123-residue (mouse or rat) or a 124-residue (human) cassette II in the cytoplasmic N-terminal region, a 20-residue cassette IV in the cytoplasmic N-terminal region, and/or a 36-residue cassette III in the cytoplasmic C-terminal region.

NBCn1 variants are found in many tissue types and are regulators of pH_i_. Additionally, NBCn1 is involved in coordinating epithelial absorption and secretion of solutes. There are 16 identified functional NBCn1 splice variants, NBCn1-A through -P. These splice variants are simplified by grouping variable regions into cassettes (Figure 6). Each functional variant contains 1 of 2 alternate N-termini, and includes or excludes previously defined cassette I, II, and/or IV in the N terminus, and/or cassette III in the C terminus (Liu et al., 2013).

NBCn1 knockout mice and genomic data have been useful in identifying tissue-specific roles for NBCn1. The transporter plays a major role in somatosensory perception based on the finding that NBCn1 knockout mice exhibit blindness and hearing loss (Bok et al., 2003; Lopez et al., 2005). NBCn1 dysfunction is also a risk factor for hypertension in humans (Ehret et al., 2011), and this link complements the phenotype of mild hypertension in the NBCn1 knockout mouse (Boedtkjer et al., 2011).

### Autoregulation

In a detailed oocyte expression study, Liu et al. (2013) discovered that each NBCn1 N-terminal region and cassette influences either membrane surface expression or activity of the transporter as measured by a pH_i_ recovery from a cellular acid load. The MEAD N terminus (Figure 6; gray N-terminus) increased NBCn1 surface expression, but not transporter activity, as compared to the MERF N terminus. Cassettes I, II, and III regulated surface expression by interacting with multiple cassettes, thereby making it difficult to assign a specific role to any single cassette. Cassette I tended to promote surface expression, but did not stimulate transporter activity. Cassette II stimulated NBCn1 activity 3.8-fold, and cassette III stimulated activity by 4.4-fold. Cassette IV had the most dramatic regulatory effect— stimulating NBCn1 activity ~11-fold, while inhibiting protein membrane abundance by 55%.

### Carbonic anhydrase

CAII binding to NBCn1 is controversial as discussed above for AE1 and NBCe1. Similar to the findings with AE1, HEK293 cells co-expressing NBCn1 and a catalytically reduced CAII mutant have a slower pH_i_ recovery from an acid load than cells expressing NBCn1 alone (Loiselle et al., 2004). However, untagged NBCn1 did not bind CAII, as described for AE1 (Piermarini et al., 2007).

### Calcineurin

Calcineurin is a calcium-dependent Ser/Thr protein phosphatase found in many cell types (Rusnak and Mertz, 2000). Calcineurin interacts with cassette II of NBCn1 based on results from a yeast 2-hybrid screen of a human skeletal muscle cDNA library (Danielsen et al., 2013). According to further mutagenesis work, this interaction is dependent on residues 94PTVVIHT (critical amino acids are underlined) within cassette II of NBCn1. Results from surface plasmon resonance further support calcineurin binding and a subsequent conformational change of cassette II of NBCn1 (Gill et al., 2014). Regarding function, raising intracellular Ca^2+^ to activate calcineurin stimulated the endogenous NBCn1-B-mediated pH_i_ recovery from an acid load in smooth muscle cells of rat mesenteric arteries (Danielsen et al., 2013). This stimulation was inhibited by the Ca^2+^ chelator BAPTA and calcineurin inhibitors FK506 and cyclosporine in a non-additive fashion. Based on the modular structure of NBCn1, cassette II is found in many variants (Boron et al., 2009) and calcineurin is expected to stimulate NBCn1-A, -B, -C, -D, and -H (Figure 6). Calcineurin stimulation of NBCe1-B is likely physiologically important by protecting the smooth muscle cells from excessive acidosis during artery contraction (Danielsen et al., 2013).

### IRBIT

As described above, IRBIT is a signaling molecule released from IP_3_ receptors when the cytosolic IP_3_ concentration rises (Ando et al., 2003). Parker et al. (2007b) demonstrated that IRBIT interacts with NBCn1-B and stimulates both the transport activity and channel-like Na^+^ conductance of NBCn1-B expressed in *Xenopus* oocytes. This IRBIT stimulation is not due to increased NBCn1-B expression (Mark D. Parker, personal communication). IRBIT co-expression in HeLa cells also stimulates NBCn1-A (Hong et al., 2013). Similar to that observed for NBCe1-B, this stimulation requires the homologous trimer of arginines (residues 56–58) within the cytoplasmic N terminus of NBCn1-A, and involves the PP-1 and WNK/SPAK pathways. Thus, IRBIT is expected to bind the other NBCn1 variants that all have the IRBIT binding domain identified in NBCe1-B.

### PIP_2_

Hong et al. have presented intriguing data that PIP_2_ also appears to stimulate NBCn1 expressed in *Xenopus* oocytes, and the stimulation requires the trimer of arginines at residues 56–58 (Hong et al., 2013). Thus, PIP_2_ regulation of BTs may not be restricted to NBCe1. However, as presented above for PIP_2_ studies on NBCe1, it is important to distinguish between the effects of PIP_2_ *per se* vs. its hydrolysis to IP_3_.

### cAMP

cAMP is a second messenger that actives PKA, thereby phosphorylating many target proteins and activating many channels and transporters, including NBCe1 described above. Applying the PKA activators forskolin or 8-bromoadenosine stimulated NBCn1 heterologously expressed in HEK293 cells (Loiselle et al., 2004). Although this inhibition was sensitive to the PKA inhibitor H89, PKA did not alter the phosphorylation state of NBCn1. Thus, phosphorylation of an intermediate NBCn1 regulatory protein may be involved.

### PDZ domain-containing proteins

Post-synaptic density protein 95 (PSD-95) is a scaffolding protein found in the post-synaptic density of neuronal dendrites. In addition to containing an SH3 domain and a catalytically inactive guanylate kinase domain, PSD-95 contains three PDZ domains that interact with other PDZ-containing proteins including the NMDA receptor (Xu, 2011). Based on co-immunoprecipitation results, PSD-95 binds to NBCn1, which contains a PDZ-binding domain (Lee et al., 2012b). Although co-expressing PSD-95 with NBCn1 did not stimulate HCO^−^_3_ transport, it did stimulate an NBCn1-associated channel-like conductance (Lee et al., 2012b). Syntrophin γ2 is another cytosolic scaffolding protein that contains a PDZ domain and binds NBCn1 (Lee et al., 2014). Co-expressing syntrophin γ2 with NBCn1 stimulated both HCO^−^_3_ transport and the NBCn1-associated channel-like conductance. Thus, PDZ domain-containing proteins can complex with NBCn1 and alter the pH physiology and/or electrical properties of excitable cells.

**Figure 7:**
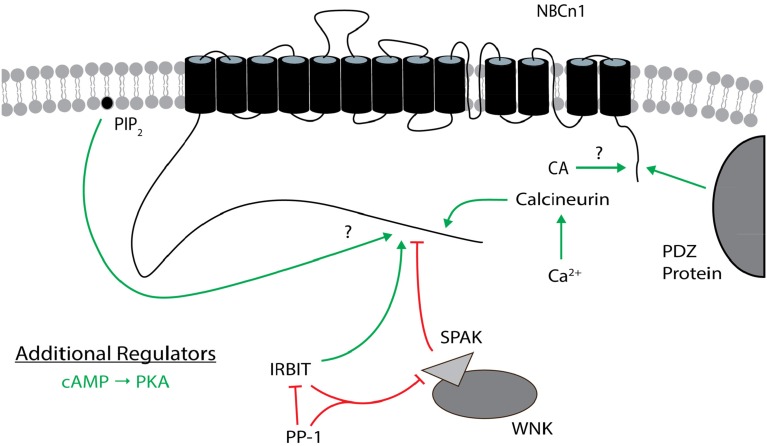
**Regulators of NBCn1**. The general position(s) of known or approximate target site(s) within NBCn1 for each regulator is/are shown. Stimulatory effects are depicted by green arrows and inhibitory effects are depicted by red lines. The direct binding of CA to NBCn1 remains controversial. PIP_2_ may stimulate itself or following PIP_2_ hydrolysis. cAMP (PKA) is an additional regulator that is not as well characterized, especially regarding its target site(s) on NBCn1. These effects involve changes in transport function and/or plasma membrane expression, and may not be the same for all variants. The many splicing cassettes and their complex combinations that are either stimulatory or inhibitory are not shown. The NBCn1 topology is based on that presented in Parker and Boron (2013). CA, carbonic anhydrase; IRBIT, inositol 1,4,5-trisphosphate (IP_3_) receptor binding protein released with IP_3_; PIP_2_, phosphatidylinositol 4,5-bisphosphate; PKA, protein kinase A; PP-1, protein phosphatase 1; SPAK, STE20/SPS1-related proline/alanine-rich kinase; WNK, with-no-lysine kinase.

Regulators of cloned NBCn1 variants are shown in Figure 7.

## NDCBE (*Slc4a8*)

**Figure 8:**
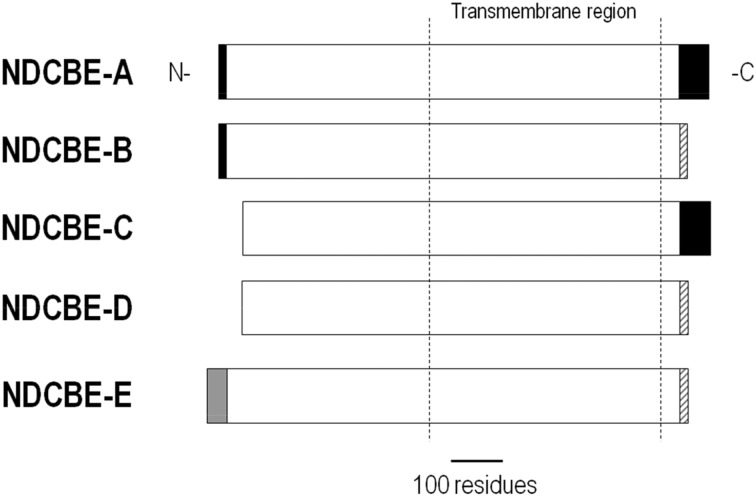
**Alignment of NDCBE variants**. NDCBE variants are identical except for differences or truncations at the N and/or C termini.

NDCBE is present in many organs, but is most abundant in the brain where it regulates pH and neuronal firing (Sinning et al., 2011). There are 5 NDCBE splice variants, NDCBE-A through -E (Figure 8). NDCBE-A and -B contain the same 16 residue N terminus, but vary in their C termini. NDCBE-C is identical to NDCBE-A, but is truncated at the N terminus by 54 residues. NDCBE-D is identical to NDCBE-B, but is truncated at the N terminus by 54 residues. NDCBE-E is identical to NDCBE-B, but the 16 residue N terminus of the B variant is replaced by a unique 43 residue N terminus. Of the 5 NDCBE variants, only the E variant has yet to be tested for function (Parker and Boron, 2013).

### Autoregulation

NDCBE-B, -D, and -E share a 17-residue C-terminal sequence (Figure 8). When expressed in *Xenopus* oocytes, NDCBE-B and -D have similar activities (Parker et al., 2008). Removing these 17 C-terminal residues of the B variant stimulated transporter activity to the level of the A variant. These findings are consistent with an AID within the different C-terminal region of NDCBE-B and -D.

### IRBIT

IRBIT, as described above, binds to and regulates other BTs including NBCe1 and NBCn1. IRBIT also binds to NDCBE-B and stimulates the transporter current (Parker et al., 2007b). However, not all NDCBE variants are stimulated by IRBIT. Parker and Boron (2013) reported the preliminary finding that IRBIT does not stimulate NDCBE-D. This variant does contain the putative IRBIT-binding RRR sequence (residues 19–21). Thus, additional domains influence IRBIT binding to BTs, as described above for NBCe1.

## NBCn2 (*Slc4a10*)

**Figure 9:**
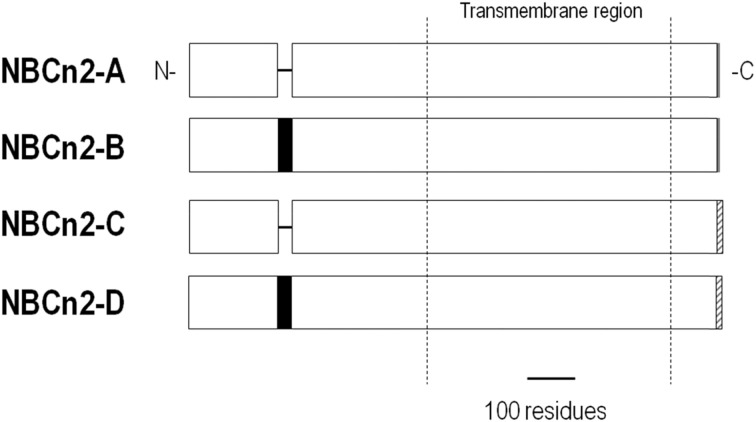
**Alignment of NBCn2 variants**. NBCn2 variants are identical except for the inclusion/exclusion of a 30-residue cassette, and/or a different C terminus.

NBCn2 is present throughout many organs. A physiological role for NBCn2 has been most apparent in the brain, where NBCn2 regulates neuronal firing. The NBCn2 knockout mouse had impaired neuronal recovery from an acid load and a higher seizure threshold (Jacobs et al., 2008). However, disruption of the NBCn2 gene in a human patient is associated with both epilepsy and mental retardation (Gurnett et al., 2008). The role of NBCn2 outside of the nervous system remains unclear (Parker and Boron, 2013). There are four NBCn2 splice variants, NBCn2-A through -D (Figure 9). NBCn2-B and -D contain a 30-residue cassette A within the N terminus. NBCn2-A and -B contain a 3-residue cassette within the C terminus, whereas NBCn2-C and -D contain a 21-residue cassette within the C terminus that has a PDZ domain.

### Autoregulation

Removing the N-terminal 92 residues of NBCn2-B increased transporter activity (Parker et al., 2007a). Thus, this region likely contains an AID, which is conserved among all the NBCn2 variants (Figure 9). However, the AID may be differentially regulated by other variable regions among the variants.

### cAMP

cAMP inhibits NBCn2-C. Applying forskolin to 3T3 cells heterologously expressing NBCn2-C (reported as a variant of NCBE) inhibited the NBCn2-mediated pH_i_ recovery from an acid load (Lee et al., 2006). The cAMP analog 8-bromo-cAMP mimicked this inhibition, whereas the PKA inhibitory fragment 14-22 stimulated transport. Disrupting the actin cytoskeleton with cytochalasin B also inhibited the NBCn2-mediated pH_i_ recovery. A potential protein that may link cytoskeleton structure to NBCn2 is the ezrin binding protein 50 (EBP50), which contains PDZ domains and also binds PKA. Indeed, EBP50 co-immunoprecipitated with transfected NBCn2-C in 3T3 cells (Lee et al., 2006). Because NBCn2-C and PKA can bind EBP50, the authors suggest that cAMP-induced NBCn2-C inhibition is an upstream regulator of EBP50 PDZ domains. Similar regulation is expected for NBCn2-D that also has a PDZ-binding domain at its C terminus.

### IRBIT

IRBIT both binds and stimulates NBCn2 (Parker et al., 2007a,b). IRBIT stimulates NBCn2 without a change in membrane abundance (Mark D. Parker, personal communication).

## Conclusions and future directions

Investigators have made considerable advances over the years in identifying and characterizing regulators of Slc4 BTs. Results from functional assays, mutagenesis, and binding studies involving immunoprecipitation and yeast 2-hybrid assays on the cloned proteins heterologously expressed in oocytes or mammalian cells have revealed a rich and diverse array of regulators and their target sites. Regulators can alter BT expression and/or function, and perhaps even transport stoichiometry. In addition to the classic second messengers (e.g., Ca^2+^, cAMP, and associated phosphorylation), there are less conventional regulators. For example, glycolytic enzymes alter the activity/expression of AE1. Autoregulatory domains influence the function of many NCBTs, and these include the N termini of NBCe1 and NBCn2, the C termini of NDCBE, and cassettes throughout NBCn1. Such autoregulation may underscore the functional significance of at least some NCBT variants. Some of these regulators exhibit crosstalk and modulate each other. IRBIT is a central player in the crosstalk of regulators already identified for NBCe1. While IRBIT stimulates NBCe1-B/C (at least partially) by removing the N-terminal AID, it also suppresses WNK/SPAK inhibition of the transporter. IRBIT also appears to interfere with Mg^2+^-induced inhibition and the PIP_2_ (or IP_3_/Ca^2+^)-induced stimulation of NBCe1, perhaps through the AID as well. Most recently, IRBIT has been shown to promote Cl^−^_i_ inhibition of NBCe1-B by unmasking a high-affinity GxxxP site.

The exciting advances already made will direct future efforts to understand better the physiological significance of regulators of Slc4 BTs. Appealing future areas of research involve the following:

- Identifying and characterizing new regulators of the cloned proteins. Other regulators and interacting proteins are expected to be identified, especially for BTs that contain PDZ-binding motifs at their C termini. Some regulators may therefore be variant-specific.
- Addressing controversies, particularly related to the importance of CAs in BT function, and the existence of a physical BT-CA metabolon.
- Determining the mechanistic basis of regulators such as glycolytic enzymes and protein 4.2 that stimulate AE1, and PIP_2_ that stimulates NBCe1. Mutagenesis data will help assign regions involved in binding. Structural information provided by biochemical techniques (such as chemical labeling and the substituted-cysteine accessibility method or SCAM), NMR, and eventual x-ray crystallography will provide insight into conformational changes that are directly responsible for altered transport function.
- Elucidating the dynamic interplay and/or crosstalk among various regulators (e.g., associated with IRBIT regulation and PIP_2_-IP_3_/Ca^2+^ regulation). These studies are linked to (iii) above. However, a challenge will be in distinguishing regulators as competitors vs. allosteric modulators, and in determining under what conditions one regulator dominates. We expect regulatory profiles to differ among variants in some cases.
- Characterizing regulation of endogenous BT function in a physiological/pathophysiological setting. This direction is highlighted by the recent evidence that IRBIT and associated regulators modulate ion and fluid secretion by ductal epithelia. Additional exciting directions include determining if high Mg^2+^_i_ or PIP_2_ hydrolysis protects against cell-damaging Na^+^_i_ and Ca^2+^_i_ overload by inhibiting NBCe1 in energy-deficient pathological conditions such as ischemia and hypoxia. For regulators of BTs that influence the electrical properties of excitable cells (e.g., heart myocytes and neurons), it will be important to assess how such regulators impact overall tissue excitability and function. Results from *in-vivo* and *in-vitro* physiological studies on specific BT knockout (KO) animals will elucidate the specific transporters or variants responsible. However, targeting a specific BT that represents a splice variant will be genetically challenging in KO studies. Conditional KO animals will be valuable in assessing the role of specific transporters/variants without confounding compensatory expression profiles.

Undoubtedly, the field will continue to evolve with new discoveries that will highlight the complexity of pH_i_ physiology. Multiple regulatory mechanisms for a single Slc4 protein and/or the presence of many different Slc4 proteins impart a cell with an intricate signaling network to orchestrate pH_i_ regulation under different conditions and stimuli.

## Author contributions

The manuscript was written and edited by IMT and MOB.

### Conflict of interest statement

The authors declare that the research was conducted in the absence of any commercial or financial relationships that could be construed as a potential conflict of interest.
